# Human Menstrual Blood-Derived Stem Cells Protect against Tacrolimus-Induced Islet Dysfunction via Cystathionine β-Synthase Mediated IL-6/STAT3 Inactivation

**DOI:** 10.3390/biom14060671

**Published:** 2024-06-08

**Authors:** Jiamin Fu, Qi Zhang, Ning Zhang, Sining Zhou, Yangxin Fang, Yifei Li, Li Yuan, Lijun Chen, Charlie Xiang

**Affiliations:** 1 State Key Laboratory for Diagnosis and Treatment of Infectious Diseases, National Clinical Research Center for Infectious Diseases, National Medical Center for Infectious Diseases, Collaborative Innovation Center for Diagnosis and Treatment of Infectious Diseases, The First Affiliated Hospital, Zhejiang University School of Medicine, Hangzhou 310003, China; 22118252@zju.edu.cn (J.F.); zhangqi2020@zju.edu.cn (Q.Z.); 22218055@zju.edu.cn (N.Z.); 11918217@zju.edu.cn (S.Z.); 12018221@zju.edu.cn (Y.F.); 1509048@zju.edu.cn (Y.L.); 2 Research Units of Infectious Disease and Microecology, Chinese Academy of Medical Sciences, Hangzhou 310003, China; 3 Innovative Precision Medicine (IPM) Group, Hangzhou 311215, China; leeyuan@ipmbiotech.com; 4 Jinan Microecological Biomedicine Shandong Laboratory, Jinan 250117, China

**Keywords:** mesenchymal stem cells, diabetes, apoptosis, transplantation

## Abstract

Diabetes imposes a huge burden worldwide. Islet transplantation is an alternative therapy for diabetes. However, tacrolimus, a kind of immunosuppressant after organ transplantation, is closely related to post-transplant diabetes mellitus. Mesenchymal stem cells (MSCs) have attracted interest for their potential to alleviate diabetes. In vivo experiments revealed that human menstrual blood-derived stem cells (MenSCs) treatment improved tacrolimus-induced blood glucose, body weight, and glucose tolerance disorders in mice. RNA sequencing was used to analyze the potential therapeutic targets of MenSCs. In this study, we illustrated that cystathionine β-synthase (CBS) contributed to tacrolimus -induced islet dysfunction. Using β-cell lines (MIN6, β-TC-6), we demonstrated that MenSCs ameliorated tacrolimus-induced islet dysfunction in vitro. Moreover, MenSC reduced the tacrolimus-induced elevation of CBS levels and significantly enhanced the viability, anti-apoptotic ability, glucose-stimulated insulin secretion (GSIS), and glycolytic flux of β-cells. We further revealed that MenSCs exerted their therapeutic effects by inhibiting CBS expression to activate the IL6/JAK2/STAT3 pathway. In conclusion, we showed that MenSCs may be a potential strategy to improve tacrolimus-induced islet dysfunction.

## 1. Introduction

In recent years, the global prevalence of diabetes has continued to increase, posing an ongoing challenge [1]. Diabetes is a chronic metabolic disease characterized by insulin deficiency/resistance. As diabetes progresses, there could be multiple organ damage such as macrovascular disease (cardiovascular disease, diabetic foot), microvascular disease (diabetic nephropathy, diabetic retinopathy, neuropathy), and diabetic ketoacidosis, leading to blindness, kidney failure, reduced overall quality of life, and even death [2]. At present, interventions for the treatment of diabetes mainly include exercise, diet control, drugs, and insulin injections. Although insulin and other oral drugs can control blood glucose, they cannot restore body homeostasis through endogenous regulation and cannot eliminate the risk of acute and chronic complications associated with diabetes. Islet transplantation is a promising method for injecting functionally isolated islet cells into the body, which can synergistically regulate blood glucose levels, maintain body homeostasis, and prevent diabetes-related complications [3]. However, islet transplantation still faces related problems, and post-transplant diabetes mellitus is one of the most prevalent complications, which is closely linked to the use of immunosuppressants [4]. Tacrolimus, a calcineurin inhibitor, is a first-line immunosuppressive drug administered after organ transplantation. Long-term use of tacrolimus can inhibit the proliferation of islet cells, promote apoptosis, and impair insulin signaling, which is key to post-transplant diabetes mellitus [5,6,7]. CBS is found in various tissues, including the liver, islets, kidneys, and pancreas, and is a key rate-limiting enzyme in the transsulfuration pathway. It co-catalyzes hydrogen sulfide alongside cystathionine gamma lyase (CSE, as a key enzyme in the sulfur transfer pathway, it can convert methionine-derived cysteine into cysteine) and 3-mercaptopyruvate sulfurtransferase (3-MST, it converts 3-mercaptopyruvate to pyruvate and hydrogen sulfide) [8]. CBS is closely associated with the occurrence and development of tumors. Overexpression of CBS inhibits cell proliferation and promotes apoptosis [9]. Additionally, hydrogen sulfide, catalyzed by CBS, inhibits the production and release of insulin [10]. Thus, CBS may disrupt blood glucose levels by affecting islet cell survival and interfering with insulin secretion.

MenSCs represent a novel category of MSCs characterized by low immunogenicity, robust differentiation, and self-renewal capabilities. Compared with other MSCs, MenSCs have unique advantages, including periodic acquisition, non-invasiveness, convenience, and no ethical disputes. Similar to other MSCs, MenSCs play an immunomodulatory role by promoting the polarization of macrophages from the M1 phenotype to the M2 phenotype, increasing the number of regulatory T cells, promoting the secretion of anti-inflammatory factors IL-10 and IL-4, and inhibiting the expression of inflammatory factors interleukin-1, tumor necrosis factor-α, and interferon-γ [11,12]. In our previous study, we found that MenSCs could reduce the blood glucose level in diabetic mice. However, the blood glucose of the mice did not return to baseline [13]. In recent years, MSC-based therapies have emerged as an alternative for diabetes treatment. In some previous studies on co-transplantation of MSCs and islet cells, the co-transplantation of MSCs and islet cells could maintain normal islet morphology. MSCs secrete various cytokines, including vascular endothelial growth factor, hepatocyte growth factor, and transforming growth factor beta, which facilitate the early rebuilding of blood vessels in islet cells and support islet cell implantation [14,15]. Moreover, MSC-secreted matrix metalloproteinases promote host-derived endothelial cell migration to islets and degrade the extracellular matrix [16,17]. Several studies on MSC treatment for diabetes have demonstrated its ability to delay diabetes onset, improve blood glucose control, reduce inflammation around islet cells, prevent autoimmune destruction, and promote pancreatic tissue regeneration [18,19,20,21,22,23]. Additionally, short-term exposure to tacrolimus does not induce toxicity or apoptosis in MSCs [24]. However, there are few studies on whether MSC can ameliorate the toxic effects of tacrolimus on islets. Moreover, the specific mechanism by which MSC exerts its therapeutic effect is still unclear.

In this study, our initial investigation focused on how MenSCs can mitigate the post-islet transplantation dysfunction induced by tacrolimus. By conducting transcriptome sequencing analysis, we found that MenSCs could down-regulate the tacrolimus-induced increase of CBS. Therefore, we hypothesized that the therapeutic benefits of MenSCs are linked to their capacity to decrease CBS expression. We further explored the possible role of CBS within the islet cell microenvironment and found that MenSCs activate the IL6/STAT3 pathway by inhibiting CBS, thereby improving islet cell survival and function. In conclusion, this study explores the feasibility of MenSCs in the field of diabetes treatment and provides a novel approach to addressing post-transplant diabetes caused by tacrolimus.

## 2. Materials and Methods

### 2.1. Cells

MenSCs were donated by the Innovative Precision Medicine Group (IPM, Hangzhou, China) and cultured as described previously [25,26]. MIN6 cells were purchased from MeisenCTCC (CTCC-001-0419, Jinhua, China), and β-TC-6 cells were purchased from Procell Life Science & Technology (CM-0322, Wuhan, China). Cells were cultured in DMEM medium (Thermo Fisher Scientific, Waltham, MA, USA), which was supplemented with 10% fetal bovine serum (FBS, 10099141c, Thermo Fisher Scientific, Auckland, New Zealand) in 5% CO_2_ at 37 °C. To create knockdown or control constructs, three shRNAs targeting the mouse CBS gene (NM_144855), along with a scrambled shRNA, were inserted into the lentiviral GV493 vector (Genechem, Shanghai, China). The target sequences are as follows: shCBS#1 (CCATCAGACGAAGTCTGCAAA), shCBS#2 (ACACTATCATTGAGCCAACTT), and shCBS#3 (CCCTATGGTCAGAATCAACAA). The MenSCs and β-cell coculture systems were as follows: MIN6 and β-TC-6 were seeded onto 6-well or 12-well plates, and MenSCs were plated on the 0.4 μm permeable polyester membrane in Transwell supports on the same day. On the second day, the transwell inserts containing MenSCs were placed above the wells seeded with MIN6 and β-TC-6 cells [27]. The culture system in the Transwell was consistent with that on the well plates. Additionally, tacrolimus (HY-13756, MedChemExpress, Monmouth Junction, NJ, USA) was dissolved in dimethyl sulfoxide (DMSO, Sigma-Aldrich, Merck, Darmstadt, Germany), and its concentration was adjusted to 800 ng/mL in DMEM complete medium [28]. Stattic, a potent STAT3 inhibitor, (HY-13818, MedChemExpress), was also dissolved in DMSO, and its concentration was adjusted to 100 μm in the DMEM complete medium. The S-adenosyl methionine (SAM) used (A2408, Sigma-Aldrich, Saint Louis, MO, USA) had a concentration of 500 μM, which was dissolved in a DMEM-complete medium [29].

### 2.2. Isolation of Mouse Islets

The mice were euthanized by full decapitation and cervical dislocation. After separating the common bile duct and clamping the ampulla of the duodenum with surgical clips, the pancreas was dissociated, and collagenase V (C9263, Sigma-Aldrich) was added (2.5 mL each) to a 50 mL centrifuge tube. An additional 2.5 mL of frozen collagenase V was included, and the mixture was allowed to digest in a 37 °C water bath for 25 min. The tissue was swirled with 10 mL of pre-cooled Hanks’ balanced salt solution (HBSS, 14175079, Thermo Fisher Scientific, New York, NY, USA) with 10% FBS until it became sandy. The samples were then centrifuged at 1000 rpm for 2 min at 4 °C, and the supernatant was discarded. The remaining pellet was resuspended in 15 mL of HBSS, followed by another centrifugation at 1000 rpm for 2 min at 4 °C. The supernatant was once again discarded, and 5 mL of Histopaque-1119 (11191, Sigma-Aldrich, Shanghai, China) was added. Afterward, 5 mL of HBSS was added, and the mixture was centrifuged at 2000 rpm for 20 min at 4 °C. The isolated islets, located between histopaque and HBSS, were collected, passed through a 70 μm cell strainer, and selected under a microscope [30]. These isolated islets were cultured in CMRL1066 (X018I0, IcellTrans, Wenzhou, China) supplemented with 10% FBS. The experiment involved using a concentration of 150 μM of aminooxyacetic acid (AOAA, C13408, Sigma-Aldrich).

### 2.3. Islet Imaging

Fluorescein diacetate (FDA, F7378, Sigma-Aldrich) and propidium iodide (PI, P4170, Sigma-Aldrich) were used to stain the islet cells, which were observed under a Leica sp8 confocal laser scanning microscope (Leica, Wetzlar, Germany) to obtain high-quality islet images.

### 2.4. Animal Model

All BALB/c mice (6-week old males) were obtained from SLAC Laboratory Animal Co., Ltd. (Shanghai, China) and maintained at the Laboratory Animal Center of Zhejiang University (ZJU-LAC). We strictly followed the instructions of the Laboratory Animal Welfare and Ethics Committee of Zhejiang University (ZJU-IACUC), under the ethics code ZJU20220393. All animals were maintained in an SPF environment with a constant temperature, 60% humidity, and adequate water and food. Each mouse was considered as a unit. To induce diabetes, mice (n = 18) were injected with 180 mg/kg streptozocin [10] (STZ, V900890, Sigma-Aldrich), which is a well-established model for inducing diabetes for studying islet transplantation [31]. Blood samples were collected from the tail vein daily for the following week to measure blood glucose levels. When nonfasting blood glucose was greater than 11.1 mmol/L for two consecutive days, the diabetic model was considered established and ready for islet transplantation [31]. Diabetic mice were euthanized using 2% isoflurane in oxygen. Following the shaving and sterilization of the left side of the back, a 5 mm incision was made. The kidney was gently exposed using forceps, and a small opening was created in the renal capsule. Each diabetic mouse received a transplant of 400 islets under the kidney capsule. After the operation, the abdominal cavity was closed, and the mice were resuscitated on a thermostatic pad. According to a random number table generated by Excel 2022 (Microsoft Corporation, Redmond, WA, USA), the experimental animals were divided into three groups (n = 6 each): the control group (NC); the tacrolimus injection group (TAC); and the MenSCs injection group (TACM). The tacrolimus group and MenSCs group received 2 mg/kg tacrolimus injections every day [32], whereas the MenSCs group was injected with MenSCs every week (8 × 10^5^ in 100 µL of normal saline) via tail vein injection [33]. Blood glucose and body weight were monitored every 2 d. Sustained blood glucose levels lower than 11.1 mmol/L after islet transplantation in mice were considered to reestablish blood glucose levels. Mice were euthanized with CO_2_ (at a rate of 30–70% replacement/min in a 10 L chamber for at least 10 min) on day 30. Venous blood and kidney tissues were then collected for further analysis. In fact, we did perform graft-bearing nephrectomy, and we observed an increase in blood glucose levels in the mice that exceeded 11.1 mmol/L the day after graft-bearing nephrectomy. The data analysts were unaware of the group allocation.

Additional information on the materials and methods is provided in Supplementary Materials and methods.

## 3. Results

### 3.1. MenSCs Proliferation and Anti-Apoptosis Were Unaffected by High Glucose and Tacrolimus Exposure

**Figure 1:**
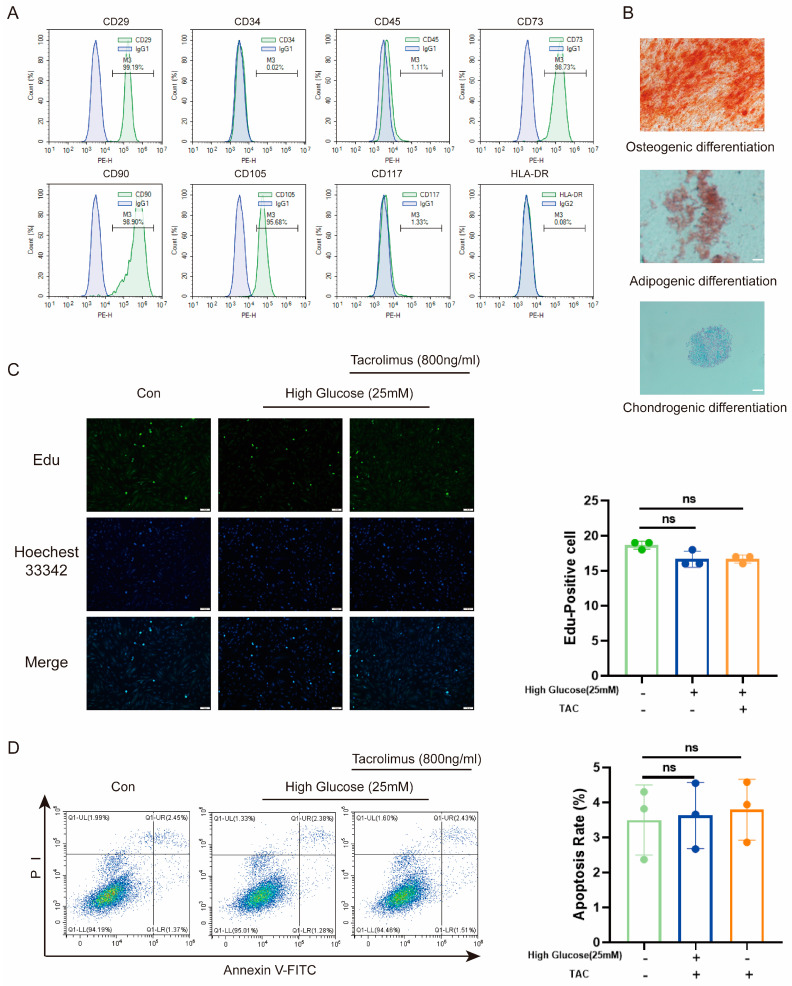
Characterization and effects of high glucose and tacrolimus (TAC) on MenSCs. (**A**) The MenSCs surface marker identification used for this experiment showed high expression of CD23, CD73, CD90, and CD105, whereas CD34, CD45, CD117, and HLA-DR were either low or nearly absent. (**B**) MenSC trilineage differentiation staining was performed at a magnification of 20× with a scale ruler of 100 μm. (**C**) Edu staining of MenSCs in MEMα, High Glucose (25 mM), and tacrolimus (800 ng/mL) at a magnification of 20× with a scale ruler of 100 μm. (**D**) Apoptosis rates of MenSCs in MEMα, high glucose (25 mM), and tacrolimus (800 ng/mL) environments were analyzed (n = 3), with statistical analysis conducted using one-way analysis of variance. Data are presented as the mean ± SD. (“ns” denotes not statistically significant).

To assess the suitability of MenSCs, we first characterized their properties. Flow cytometry revealed low expression of CD34, CD45, CD117, and HLA-DR, along with high expression of CD29, CD73, CD90, and CD105 (Figure 1A). Moreover, a three-lineage differentiation assay demonstrated their capability to differentiate into osteoblasts, adipogenic cells, and chondrogenic cells (Figure 1B). These indicated their low immunogenicity and multilineage differentiation potential. Given the high glucose (25 mM) [34] environment in diabetes and the prolonged use of tacrolimus following transplantation, we investigated the effects of high glucose and tacrolimus stimulation on proliferation of MenSCs and resistance to apoptosis. Results from 5-ethynyl-2′-deoxyuridine (Edu) staining (Figure 1C) and flow cytometry (Figure 1D) confirmed that MenSCs retained their proliferation and anti-apoptosis abilities under conditions of high glucose and tacrolimus exposure. Overall, these results support the suitability of MenSCs for transplantation into animal models.

### 3.2. MenSCs Ameliorated Tacrolimus-Induced Islet Dysfunction after Transplantation In Vivo

**Figure 2:**
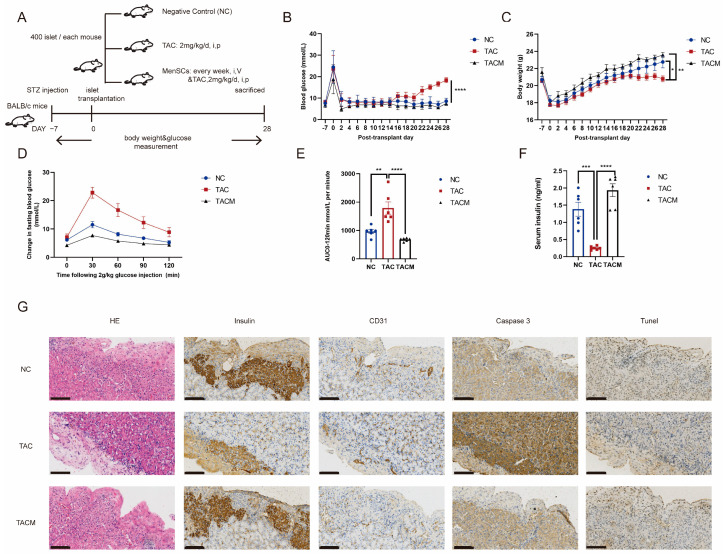
MenSCs improve tacrolimus (TAC)-induced islet dysfunction after transplantation. (**A**) The establishment of a mouse diabetes model and monitoring of blood glucose and body weight after islet transplantation were divided into NC, TAC, and TACM groups. (**B**) Results of nonfasting blood glucose measurements in mice. (**C**) Mouse weight measurements. (**D**) Mouse intraperitoneal glucose tolerance tests. (**E**) Mouse area under the curve (AUC) results. (**F**) Serum insulin levels after 30 min of glucose tolerance induction in mice. (**G**) Monitoring of islet transplantation in mice, including IHC (immunohistochemistry) staining for insulin, CD31, Caspase3, and Tunel expression at a 20× magnification with a scale ruler of 100 microns (n = 6). Statistical analysis was performed using one-way analysis of variance. Data are presented as the mean ± SD. (* *p* < 0.05, ** *p* < 0.01, *** *p* < 0.001, and **** *p* < 0.0001).

Our in vivo study involved establishing a diabetic mouse model and performing islet transplantation, as shown in Figure 2A (details are provided in Section 2). After inducing hyperglycemia using streptozotocin, all mice achieved random blood glucose levels exceeding 20 mmol/L. Islet transplantation occurred 1 week after achieving stable blood glucose levels. Notably, the MenSCs co-transplantation group exhibited more favorable blood glucose regulation following islet transplantation. In contrast, the tacrolimus group initially showed a return to baseline blood glucose levels after islet transplantation, but glucose levels subsequently rose with continued tacrolimus usage, leading to a blood glucose imbalance at 2–3 weeks (Figure 2B). Additionally, the MenSCs co-transplantation group displayed more significant post-transplantation weight gain compared to the tacrolimus group, which ceased gaining weight at 2–3 weeks (Figure 2C). Glucose tolerance testing revealed impaired glucose load in the tacrolimus group, with significantly increased blood glucose levels at 30 min. In contrast, the MenSCs co-transplantation group displayed minimal changes in fasting blood glucose at 30 min, nearly returning to baseline levels by 120 min (Figure 2D). A similar trend was observed in the area under the curve, with the MenSCs co-transplantation group showing improved glucose clearance while the tacrolimus group exhibited significant impairment (Figure 2E). Subsequent measurement of serum insulin levels at 30 min revealed significantly lower levels in the tacrolimus group, aligning with the results of the glucose tolerance test. The tacrolimus group had insufficient serum insulin levels to effectively respond to the glucose challenge, indicating a glucose imbalance. In contrast, the MenSCs co-transplantation group exhibited sufficient insulin secretion in response to glucose stimulation, maintaining homeostasis (Figure 2F). Immunohistochemical staining of the islet transplantation site showed that insulin secretion in the tacrolimus group was significantly decreased, accompanied by increased apoptosis. The staining for caspase3 and Tunel showed notably stronger signals compared to the NC group. Simultaneously, the vascularization was compromised, as indicated by the weaker CD31 staining in the tacrolimus group, whereas these markers exhibited substantial improvement in the MenSCs group (Figure 2G).

### 3.3. CBS Inhibited β-Cell Viability, Anti-Apoptotic and GSIS, Whereas MenSCs Inhibited β-Cell CBS Levels

**Figure 3:**
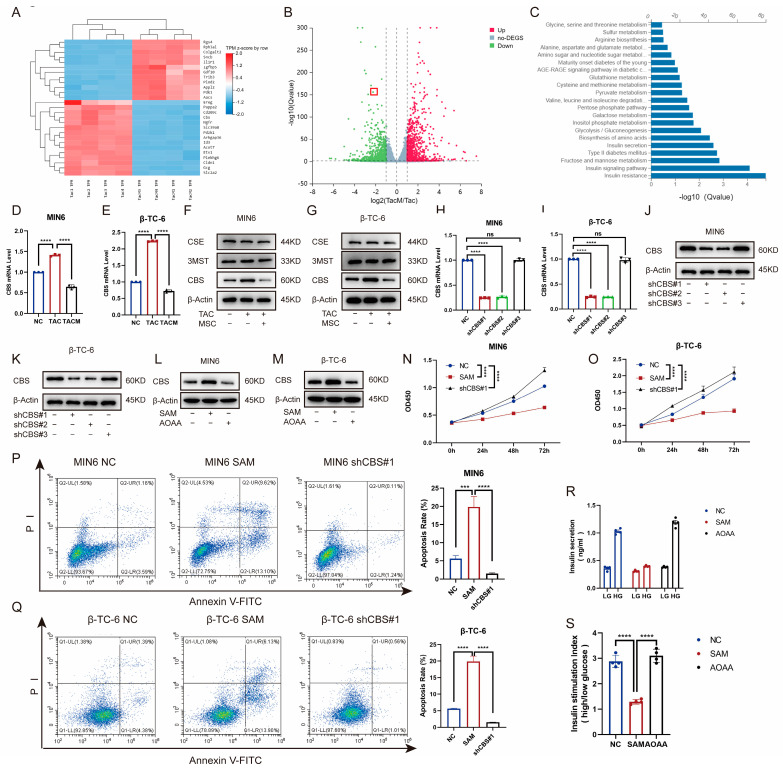
MenSCs inhibit β-cell CBS levels to promote β-cell survival and improve glucose tolerance. (**A**) Cluster heatmap of differential gene expression. (**B**) Volcano plots of the different gene expression levels. (**C**) GO analysis of significantly enriched genes. (**D**,**E**) Analysis of CBS mRNA expression in MIN6 and β-TC-6 cells treated with normal, TAC, and TACM (n = 3). (**F**,**G**) Protein expression of CBS, CSE, and 3MST in MIN6 and β-TC-6 cells under normal, TAC, and TACM treatment (n = 3). (**H**–**K**) MIN6, CBS knockdown efficiency in β-TC-6 (n = 3). (**L**,**M**) Protein expression of CBS in MIN6 and β-TC-6 cells treated with normal, SAM, and AOAA. Original images can be found in Supplementary Materials. (**N**,**O**) Cell viability of MIN6 and β-TC-6 cells in normal, SAM (S-adenosylmethionine), and CBS knockdown conditions at 72 h (n = 6). (**P**,**Q**) Apoptosis ratio of MIN6 and β-TC-6 cells in normal, SAM, and CBS knockdown conditions (n = 3). (**R**,**S**) Islet cells in GSIS under normal, SAM, and AOAA conditions (n = 4). Statistical analysis was conducted using one-way analysis of variance. Data are presented as the mean ± SD. (*** *p* < 0.001, and **** *p* < 0.0001; “ns” denotes not statistically significant).

To explore the mechanisms through which MenSCs ameliorate tacrolimus-induced islet dysfunction, we compared transcriptome levels between the tacrolimus and MenSCs groups using RNA-seq. The expression of the CBS genes related to cell proliferation and survival differed significantly between the two groups. Catalytic production of hydrogen sulfide plays an important role in the occurrence and development of diabetes (Figure 3A–C). Furthermore, we examined CBS, CSE, and 3-MST expression in MIN6 and β-TC-6 islet cell lines. Although tacrolimus promoted CBS expression at the RNA and protein levels, MenSCs significantly inhibited CBS expression, with no significant difference between CSE and 3-MST (Figure 3D–G). To investigate the effect of CBS on islet cells, we introduced lentivirus-mediated CBS knockdown in MIN6 and β-TC-6 cells, confirming stable CBS knockdown through real-time PCR and western blotting (Figure 3H–K). We also evaluated the expression of CBS in MIN6 and β-TC-6 cells following stimulation with SAM (a CBS activator) and AOAA (a CBS inhibitor). SAM was found to promote CBS expression in β-cells, whereas AOAA inhibited CBS expression in β-cells (Figure 3L–M). The CCK8 assay showed that SAM inhibited the viability of MIN6 and β-TC-6 cells, whereas CBS knockdown significantly increased cell viability (Figure 3N–O). CBS knockdown ameliorated tacrolimus-induced reductions in cell apoptosis. In contrast, SAM significantly induced apoptosis (Figure 3P–Q). Additionally, we evaluated GSIS in islet cells. SAM significantly downregulated GSIS expression in islet cells, whereas AOAA restored GSIS expression, which rendered islet cells more proficient in coping with high-glucose environments (Figure 3R–S). These findings suggest that CBS plays a critical role in tacrolimus-induced islet dysfunction and that MenSCs may attenuate tacrolimus-induced islet dysfunction by reducing CBS.

### 3.4. MenSCs-Secreting Factors Inhibited the Increase of CBS Level Induced by Tacrolimus to Improve the Viability and Anti-Apoptosis of Islet Cells

**Figure 4:**
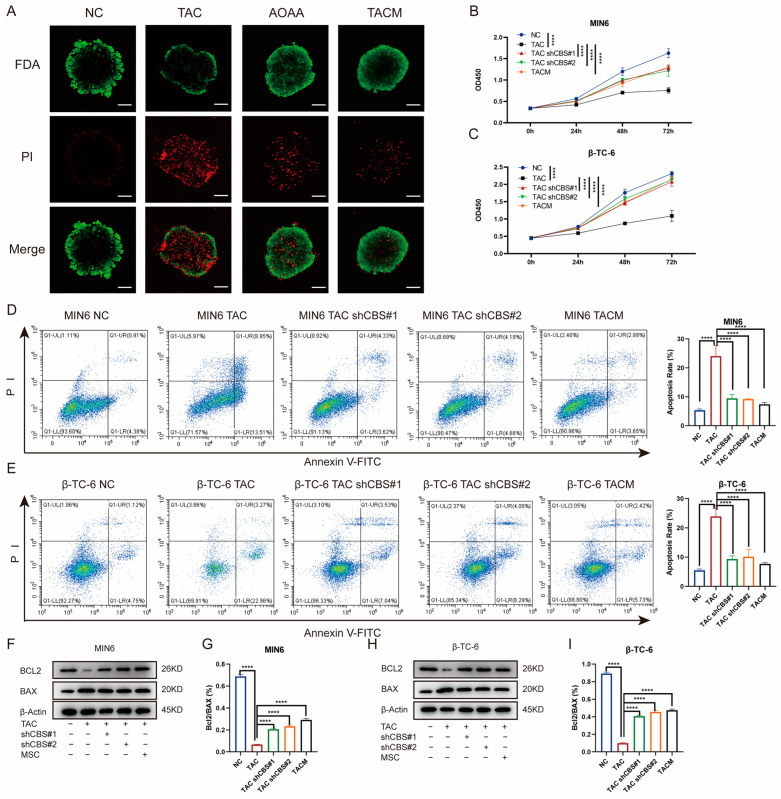
Inhibition of CBS expression by MenSCs improved the survival rate of islet cells. (**A**) FDA/PI staining of islet cells under normal, TAC, AOAA, and TACM treatments, where FDA (green) represents live cells and PI (red) represents dead cells (n = 6), observed at a 20× magnification with a scale ruler of 100 μm. (**B**,**C**) Cell viability of MIN6 and β-TC-6 cells under normal, TAC, CBS knockdown, and TACM treatment at 72 h (n = 6). (**D**,**E**) Apoptosis rate of MIN6 and β-TC-6 cells under normal, TAC, CBS knockdown, and TACM treatment (n = 3). (**F**–**I**) Protein expression of BCL2 and BAX in MIN6 and β-TC-6 cells under normal, TAC, CBS knockdown, and TACM treatment, including the calculation of the BCL2/BAX ratio (n = 3). Original images can be found in Supplementary Materials. Statistical analysis was performed using one-way analysis of variance. Data are presented as the mean ± SD. **** *p* < 0.0001).

To further investigate the impact of MenSCs inhibiting CBS expression on islet cell survival, we initiated a series of assessments. First, we determined the percentage of viable islet cells by staining them with fluorescein diacetate and PI. Tacrolimus significantly reduced the percentage of viable islet cells. However, AOAA and MenSCs treatments significantly restored islet cell viability (Figure 4A). Our investigation also encompassed CCK8 assays, which revealed that tacrolimus significantly inhibited the proliferation of MIN6 and β-TC-6 cells. Nonetheless, CBS knockdown and MenSCs treatment significantly restored the cell viability of MIN6 and β-TC-6 cells (Figure 4B,C). Employing flow cytometry analysis, we observed that tacrolimus significantly induced apoptosis in MIN6 and β-TC-6 cells. Conversely, CBS knockdown and MenSCs intervention significantly inhibited tacrolimus-induced apoptosis, significantly improving the survival of islet cells in the tacrolimus environment (Figure 4D,E). Simultaneously, we detected the expression of the anti-apoptotic factor BCL-2 and the pro-apoptotic factor BAX in islet cells through western blotting. Tacrolimus significantly reduced the BCL-2/BAX ratio and induced cell apoptosis, whereas CBS knockdown and MenSCs treatment restored the BCL-2/BAX ratio (Figure 4F–I). Taken together, these findings emphasize the remarkable ability of MenSCs to mitigate islet cell proliferation and apoptosis, underline the advantageous impact of CBS knockdown within islet cells on cell survival in a tacrolimus-rich environment, and shed light on the protective role of MenSCs in islet cells through the inhibition of CBS expression.

### 3.5. MenSCs-Secreting Factors Inhibited the Increase of CBS Induced by Tacrolimus to Improve GSIS and Glycolysis of Islet Cells

**Figure 5:**
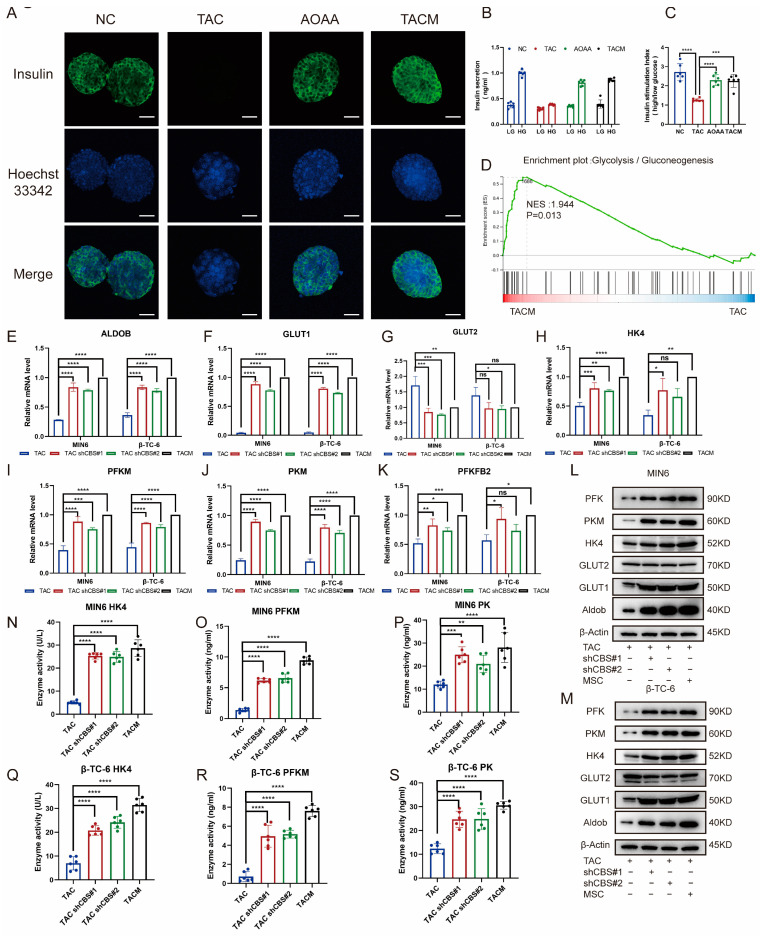
MenSCs inhibited CBS expression to improve GSIS and glycolysis in islet cells. (**A**) Insulin secretion of islet cells under normal, TAC, AOAA, and TACM treatments (n = 6) was observed at 20× magnification with a scale ruler of 100 μm. (**B**,**C**) GSIS of islet cells under normal, TAC, AOAA, and TACM conditions (n = 6). (**D**) GSEA of the glycolysis/gluconeogenesis pathway after beta-TC-6 was treated with TAC and TACM. (**E**–**K**) Evaluation of glycolysis-related genes (RNA expression of GLUT1, GLUT2, HK4, PFKM, PKM, and PFKFB2) in MIN6 and β-TC-6 cells in TAC, CBS knockdown, and TACM treatment (n = 3). (**L**,**M**) Protein expression of Aldob, GLUT1, GLUT2, HK4, PKM, and PFK in MIN6 and β-TC-6 cells in TAC, CBS knockdown, and TACM conditions. Original images can be found in Supplementary Materials. (**N**–**S**) Detection of HK4, PK, and PFKM expression in the supernatant of one million MIN6 and one million β-TC-6 cells after treatment with TAC, CBS knockdown, or TACM for 24 h using ELISA (n = 6). Statistical analysis was conducted using one-way analysis of variance. Data are presented as the mean ± SD. (* *p* < 0.05, ** *p* < 0.01, *** *p* < 0.001, and **** *p* < 0.0001; “ns” denotes not statistically significant).

Through an examination of insulin staining in islet cells, it was evident that tacrolimus stimulation significantly impaired insulin secretion, almost completely suppressing it. The addition of AOAA and MenSCs, which acted to inhibit CBS, notably improved insulin secretion (Figure 5A). GSIS is an important ability of islet cells to cope with high glucose environments. By detecting GSIS stimulated by tacrolimus, we found that the GSIS of islet cells was significantly impaired after tacrolimus stimulation; insulin secretion was significantly inhibited in a high-glucose environment; whereas the GSIS of islet cells was significantly restored after adding MenSCs and AOAA. The inhibition of CBS expression significantly improved GSIS in islet cells (Figure 5B,C). Transcriptome sequencing revealed that tacrolimus inhibited the expression of glycolysis-related genes, whereas MenSCs significantly restored the expression of related genes. Glycolysis is an important step in GSIS and plays an important role in the progression of diabetes (Figure 5D). We examined the expression of glycolysis-related genes under the conditions of CBS knockdown and MenSCs intervention using real-time PCR. We found that CBS knockdown and MenSCs significantly promoted the expression of aldolase B (Aldob), glucose transporter 1 (GLUT1), hexokinase (HK4), phosphofructokinase (PFKM), pyruvate kinase (PKM), and 6-phosphofructo-2-kinase/fructose-2,6-bisphosphatase 2 (pfkfb2) (Figure 5E–K). Western blot analysis further confirmed that the inhibition of CBS expression upregulated the protein levels of several glycolytic genes in islet cells (Figure 5L–M). We further examined the activities of three key enzymes involved in glycolysis. Using ELISA, we found that tacrolimus stimulation significantly attenuated HK4, PFK, and PK. However, the inhibition of CBS led to a marked increase in the activities of HK4, PFK, and PK (Figure 5N–S).

These results indicate that tacrolimus impairs GSIS and the expression of glycolysis-related genes in islets. MenSCs can inhibit the expression of CBS to repair impaired GSIS and upregulate the activities of glycolysis-related genes and key enzymes in islets.

### 3.6. CBS Exerts Its Effect by Regulating STAT3 Phosphorylation through IL-6

**Figure 6:**
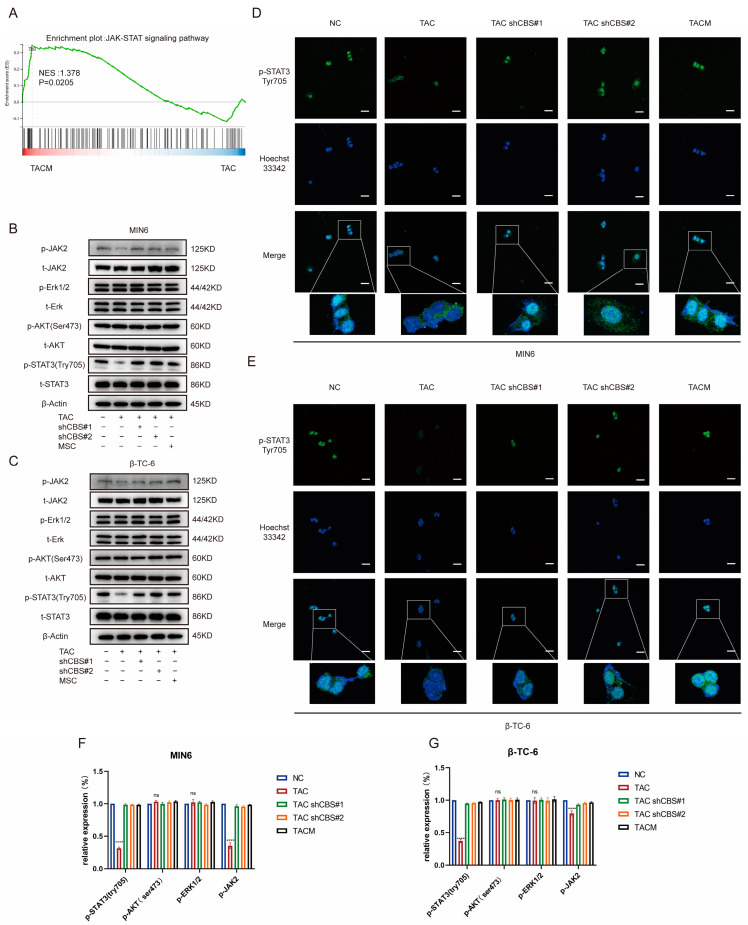
MenSCs inhibit CBS expression and activate STAT3 phosphorylation in the nucleus. (**A**) GSEA of the JAK-STAT signaling pathway after β-TC-6 was treated with TAC and TACM. (**B**,**C**) Protein expression levels of t-STAT3, p-STAT3 (Try705), t-AKT, p-AKT (Ser473), t-Erk, p-Erk1/2, t-JAK2, and p-JAK2 in MIN6 and β-TC-6 cells under normal conditions, TAC treatment, CBS knockdown, and TACM treatment. Original images can be found in Supplementary Materials. (**D**,**E**) Immunofluorescence analysis of p-STAT3 (Try705) localization in MIN6 and β-TC-6 cells under normal, TAC, CBS knockdown, and TACM treatment. (**F**,**G**) The relative expression (%) of p-STAT3 (Try705), p-AKT (Ser473), p-Erk1/2, and p-JAK2. Magnification: 63×, scale: 100 μm. (**** *p* < 0.0001; “ns” denotes not statistically significant).

**Figure 7:**
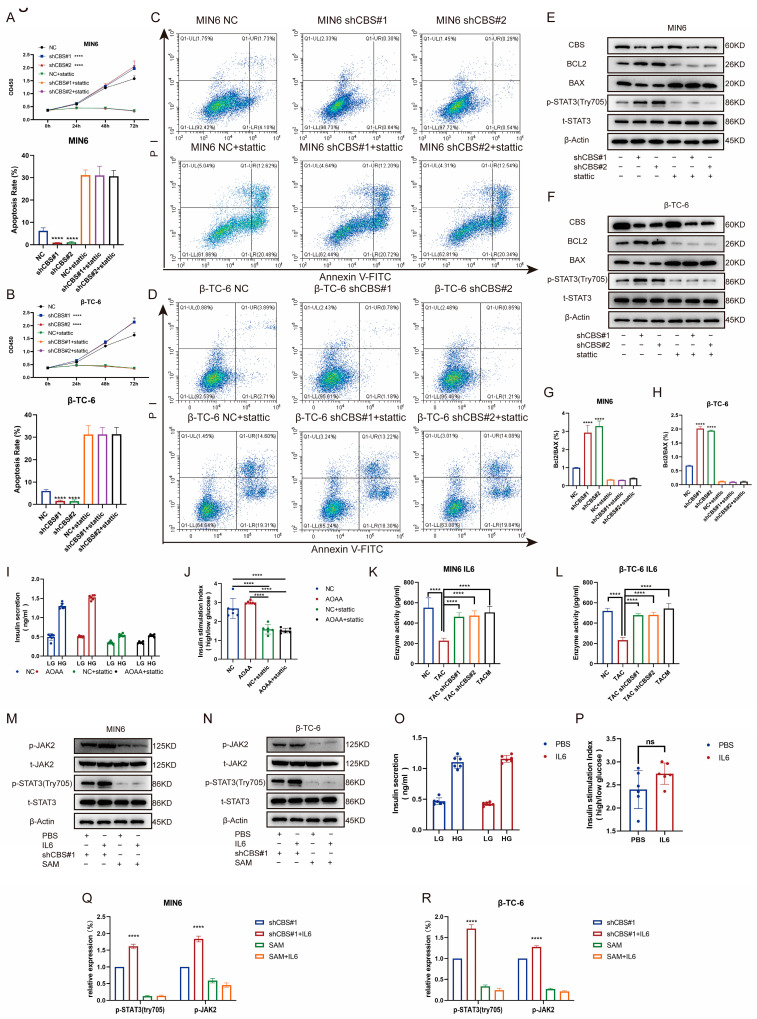
Inhibition of CBS expression by MenSC improved islet cell survival, GSIS, and glycolytic flux by up-regulating IL6 levels to activate STAT3 phosphorylation. (**A**,**B**) The CCK8 assay was used to detect the cell viability of MIN6 and β-TC-6 cells with CBS knockdown in the presence of Stattic treatment. A statistical difference occurred at 72 h, n = 6. (**C**,**D**) Ratio of apoptosis in CBS knockdown MIN6 and β-TC-6 cells in response to Stattic treatment (n = 3). (**E**–**H**) Expression levels of t-STAT3, p-STAT3 (Try705), BCL2, and BAX proteins in MIN6 and β-TC-6 cells with CBS knockdown treated with Stattic, along with the calculated BCL2/BAX ratio, n = 3. (**I**,**J**) GSIS assessment of islet cells under Stattic treatment, n = 6. (**K**,**L**) Evaluation of IL-6 expression in one million MIN6 and one million β-TC-6 cells treated with TAC, CBS knockdown, and TACM for 24 d. Supernatants were collected and analyzed by ELISA (n = 6). (**M**,**N**) Comparison of the protein expressions of t-STAT3, p-STAT3 (Try705), t-JAK2, and p-JAK2 between MIN6 and β-TC-6 cells with CBS knockdown and SAM stimulation under IL6 treatment, n = 3. Original images can be found in Supplementary Materials. (**O**,**P**) Comparison of GSIS in islet cells with CBS knockdown and SAM stimulation under IL6 treatment, n = 6. (**Q**,**R**) The relative expression (%) of p-STAT3 (Try705) and p-JAK2. A one-way analysis of variance was used for the statistical analysis. Data are presented as the mean ± SD. (**** *p* < 0.0001; “ns” denotes not statistically significant).

Studies have shown that the regulation of insulin expression primarily involves AKT, ERK, STAT3, and other pathways. Gene set enrichment analysis based on RNA sequencing demonstrated that MenSCs ameliorated tacrolimus-induced islet damage mainly through the JAK/STAT3 pathway (Figure 6A). We found that tacrolimus treatment significantly downregulated p-STAT3 (Tyr705), whereas CBS knockdown and MenSCs treatment significantly restored p-STAT3 (Tyr705) activity. In contrast, p-AKT (Ser473) and p-ERK1/2 exhibited no significant differences (Figure 6B,C,F,G). Further substantiating these findings, we conducted immunofluorescence analysis, which underscored that tacrolimus treatment suppressed STAT3 phosphorylation into the nucleus, whereas MenSCs or CBS knockdown acted to activate STAT3 phosphorylation in the nucleus by inhibiting CBS activity (Figure 6D,E). We found that CBS knockdown promoted islet cell proliferation and inhibited apoptosis, which were reversed by the STAT3 inhibitor, Stattic. CCK8 assays showed that Stattic significantly inhibited the proliferation ability of CBS knockdown cell lines MIN6 and β-TC-6 (Figure 7A,B). Moreover, Stattic significantly reduced the BCL2/BAX ratio, as evidenced by western blotting (Figure 7E–H). Flow cytometry analysis showed that Stattic significantly induced apoptosis in MIN6 and β-TC-6 cells (Figure 7C,D). Simultaneously, we examined the GSIS of islet cells exposed to Stattic, which significantly impaired the insulin secretion ability of islet cells in a high-glucose environment (Figure 7I,J). ELISA findings showed that MenSCs significantly stimulated the production of IL6 in islet cells by inhibiting the expression of CBS (Figure 7K,L). Western blotting revealed that p-STAT3 (Tyr705) was activated by the upregulation of p-JAK2 activity in response to IL6 stimulation (Figure 7M,N,Q,R). Finally, we examined GSIS in islet cells upon IL6 stimulation and found that IL6 stimulation increased GSIS expression in islet cells (Figure 7O,P). These results indicated that CBS mediates the stimulatory effect of IL6 on GSIS in islet cells. In conclusion, our results showed that CBS affects islet cell survival, GSIS, and glycolytic flux by regulating the STAT3 pathway.

## 4. Discussion

In this study, we showed that tacrolimus not only affects insulin secretion but is also highly associated with the proliferation and apoptosis of islet cells. Through RNA-seq analysis, we found that MenSCs inhibited tacrolimus-induced elevation of CBS expression. GSIS is crucial for managing high glucose levels, and tacrolimus impairs GSIS. However, MenSCs treatment restores tacrolimus-induced GSIS impairment by inhibiting CBS expression. MenSCs restored the expression of glycolytic key enzymes: HK4, PFK, and PK. Additionally, MenSCs promote the anti-apoptosis ability of β cells by inhibiting the expression of CBS. We provide evidence that MenSCs promote STAT3 activation by inhibiting CBS expression, leading to improved cell viability and glucose tolerance in β-cells. The influence of CBS on STAT3 regulation is mediated by IL6, a cytokine crucial for insulin secretion and glucose regulation [35,36,37]. Our study showed that moderate IL6 stimulation can augment the GSIS of β-cells and improve their glucose tolerance.

Tacrolimus is a well-documented factor in the development of post-transplant diabetes mellitus. Previous studies on post-transplant diabetes mellitus caused by tacrolimus have mainly focused on the effects of tacrolimus on genes related to insulin secretion, resistance, and function [38,39]. To explore the underlying mechanisms, because of the limited number of isolated islet cells and the limited survival time in vitro, we used MIN6 and β-TC-6 to study the underlying mechanisms in vitro [40]. In the future, we will use single-cell sequencing and other advanced methods to investigate how stem cells affect islet cell function in vivo. In RNA-seq, we identified the key target gene, CBS, for MenSCs to exert therapeutic effects. In terms of other differentially expressed genes identified in our RNA-seq analysis, MenSCs promoted insulin-like growth factor-binding protein 5, a secreted protein that can activate early growth response 1 and promote 6-phosphofructo-2-kinase/fructose-2,6-bisphosphatase 3 expression, thereby enhancing glycolysis [41]. Insulin-like growth factor binding protein 5 also regulates endoplasmic reticulum stress to alleviate glucose tolerance in diabetic mice [42]. MenSCs also altered the expression of interleukin 1 receptor type I, glucagon, and pyruvate dehydrogenase kinase 1, which are also involved in the development of diabetes. We intend to explore these effects further in future studies. GSIS involves glucose uptake, glycolysis, and adenosine triphosphate production [43]. Glycolysis is the first step in the coupling of glucose catabolism and insulin secretion and is primarily controlled by three key enzymes: HK4, PFK, and PK. Impaired glucose tolerance is strongly associated with the development and progression of diabetes [44,45,46], yet the specific mechanisms controlling islet cell glycolysis remain poorly understood. In this study, we found that downregulation of CBS was required for the maintenance of glycolytic flux in β-cells, whereas tacrolimus-induced CBS elevation downregulated glycolytic genes, an effect countered by MenSCs treatment. Our results suggest that MenSCs treatment provides a potential strategy for addressing tacrolimus-induced post-transplant diabetes mellitus.

The expression trend of CBS in different tissues is inconsistent. For instance, CBS expression is significantly increased in thyroid and ovarian malignancies, whereas it is significantly decreased in hepatocellular carcinoma [47,48]. The regulatory effects of CBS on cell growth vary among tissues. Overexpression of CBS can inhibit the proliferation and anti-apoptotic ability of hepatocellular carcinoma, whereas inhibition of CBS in chronic myeloid leukemia can promote cell apoptosis and reduce proliferation [9,49]. Previous studies on MSCs and sulfur transfer pathways have focused on hydrogen sulfide, CSE. These elements mediate mesenteric artery dilatation through hydrogen sulfide release, promote CACNA1H-mediated calcium influx through CSE, and control mesenteric artery dilatation through hydrogen sulfide release. However, there have been few studies on the regulation of the CBS by MSCs [49,50]. Our study suggests that CBS, as a target for MenSC regulation, could provide new options for disease treatment using MenSCs.

Previous studies have shown that tacrolimus can regulate the PI3K-AKT-mTOR pathway to regulate the insulin signaling pathway in β-cells. AKT is an important pathway in cell growth regulation that affects cell proliferation and apoptosis [51,52,53]. In this study, we determined that CBS interfered with β-cell GSIS and glycolytic flux by inhibiting STAT3 activity, whereas AKT and Erk pathways were not affected by tacrolimus. As a transcription factor, STAT3 is involved in regulating cell proliferation, survival, differentiation, and angiogenesis. STAT3 phosphorylation alleviates diabetes-related lesions [54]. Moreover, STAT3 can inhibit hepatic lipogenesis and gluconeogenesis while promoting glycogen synthesis [55]. In future studies, we will investigate how downstream genes regulated by STAT3 affect the physiological processes of islets.

MSCs are well-known for their protective effects through paracrine mechanisms. In our previous study, we demonstrated that MenSCs promote cell survival by secreting interleukin-6, hepatocyte growth factor, and other growth factors [26]. In this study, we observed that MenSCs can mitigate the toxic effects of tacrolimus on β cells through a specific pathway, but we did not verify whether this protective function also benefits from the paracrine pathway. Identifying the specific cytokines involved in the therapeutic effects of MSCs remains a common challenge in paracrine research on stem cells. In our future work, we will focus on further elucidating the mechanisms of MenSCs’ protective effects. This series of works contributes to paving the way for research on the paracrine pathways of stem cells and pancreatic islet transplantation.

## 5. Conclusions

Taken together, this study sheds light on how MenSCs regulate β-cell viability and insulin secretion by affecting CBS expression. Specifically, CBS depletion can trigger IL6 release in β-cells, activating STAT3 and improving cell viability and insulin secretion. This study introduces novel avenues of exploration for tacrolimus-induced post-transplant diabetes mellitus and identifies MenSCs as a potential treatment for tacrolimus-induced islet dysfunction and post-transplant diabetes mellitus.

## Data Availability

The raw data supporting the conclusions of this article will be made available by the authors upon request.

## Supplementary Materials

The supporting information can be downloaded at: https://www.mdpi.com/article/10.3390/biom14060671/s1. S2.5 Intraperitoneal glucose tolerance tests. S2.6 Glucose-stimulated insulin secretion (GSIS). S2.7 Edu cell proliferation. S2.8 Cell viability assay. S2.9 Western blotting. S2.10 Quantitative PCR. S2.11 Flow cytometric analyses. S2.12 Immunofluorescence. S2.13 ELISA. S2.14 Immunohistochemistry. S2.15 Statistical analysis.
