# Supplementary material for: Human Menstrual Blood-Derived Stem Cells Protect against Tacrolimus-Induced Islet Dysfunction via Cystathionine β-Synthase Mediated IL-6/STAT3 Inactivation
Source: Biomolecules. 2024 Jun 8;14(6):671. doi: 10.3390/biom14060671 (PMC11201965; doi:10.3390/biom14060671)
Supplement: Supplementary file 1 [file biomolecules-14-00671-s001.zip › biomolecules-2993506-supplementary.pdf]

Supplementary materials and methods

S2.5 Intraperitoneal glucose tolerance tests

Intraperitoneal glucose tolerance tests were performed on day 28 after transplantation to further monitor islet function. Mice were fasted overnight, and each mouse was intraperitoneally injected with glucose (2 g/kg). Blood glucose levels were monitored at 0, 30, 60, 90, and 120 min after transplantation. Venous blood was collected after 30 min for insulin level analysis.

S2.6 Glucose-stimulated insulin secretion (GSIS)

In each group, 50 islets were incubated with Krebs-Ringer Bicarbonate HEPES (KRBH) buffer, composed of NaCl (140 mM), KCl (3.6 mM), NaHCO3 (2 mM), NaH2PO4 (0.5 mM), MgSO4 (0.5 mM), CaCl2 (1.5 mM), and HEPES (10 mM), with a pH of 7.4. This buffer also contained 0.1% BSA supplemented with 2.8 mM glucose. The incubation took place at 37 °C with 5% CO2 for 1 h. Next, the supernatant was collected after another 1 h incubation with fresh 2.8 mM glucose. Additionally, the supernatants collected after incubation with 16.7 mM glucose for 1 h were preserved at -20°C for subsequent Enzyme-Linked Immunosorbent Assay (ELISA) analysis[29].

S2.7 Edu cell proliferation

MenSCs were inoculated in 6-well plates (Corning) according to the manufacturer's instructions and then stained with BeyoClick Edu-488 (C0071S, Beyotime, Shanghai, China) for 2 h. Fluorescence was measured at 488 and 346 nm using an OLYMPUS IX83-DP70 fluorescence microscope (Olympus Corporation, Tokyo, Japan).

S2.8 Cell viability assay

Cell viability was measured using a CCK-8 colorimetric kit according to the manufacturer's instructions (C0043, Beyotime, Shanghai, China).

S2.9 Western blotting

Western blotting was performed as previously described[25, 26, 32]. Antibodies against specific proteins were sourced from different manufacturers: β-actin (dilution: 1:2000,#4970), BAX (dilution：1：1000, #2772), AKT (dilution: 1:1000,#4691), Phospho-AKT (p-AKT, dilution: 1:1000, #4060), Erk1/2 (dilution: 1:1000,#4695), Phospho-Erk1/2 (pErk1/2, dilution: 1:1000,#4370), CSE (dilution: 1:1000,#19689), CBS (dilution: 1:1000,#14782), STAT3 (dilution: 1:1000,#9139), Phospho-Stat3 (pSTAT3, dilution: 1:1000,#9145), JAK2 (dilution: 1:1000,#3230), Phospho-Jak2 (pJAK2, dilution: 1:1000,#3771), PKM (dilution: 1:1000,#3198), Anti-rabbit IgG (dilution: 1:5000,#7074), and anti-mouse IgG (dilution: 1:3000,#7076) were obtained from Cell Signaling Technology (Danvers, MA, USA). The antibody against 3-MST (dilution: 1:1000,ab154514) was purchased from Abcam. Antibodies against BCL-2 (dilution: 1:1000,A19693) and PFKM (dilution: 1:1000,A3671) were acquired from ABclonal (Wuhan, China). The antibody against Aldolase B (Aldob, dilution: 1:200, sc-393278) was purchased from Santa Cruz Biotechnology (Shanghai, China). Furthermore, antibodies against GCK (HK4, dilution: 1:1000,19666), GLUT2 (dilution: 1:1000,20436), and

GLUT1 (dilution: 1:1000,21829) were purchased from Proteintech (Wuhan, China).

S2.10 Quantitative PCR

Quantitative PCR was performed as previously described[25, 26, 32]. The relative mRNA expression was analyzed using the **ΔΔct** method. The primers used for real-time PCR were as follows: Mouse β-actin: Forward 5′-TGTGGATCGGTGGCTCCATCCT-3,′ Reverse 5′-AAACGCAGCTCAGTAACAGTCCGC-3′; Mouse CBS: Forward 5′-GGAAAATTGGGAACACCCCTAT-3,′ Reverse 5′-CCACCCGCATTGAAGAACTCA-3′; Mouse CSE: Forward 5′-GAGCAGTTCCATCTCCTATTGA-3,′ Reverse 5′-GGCAGCCCAGGATAAATAAC-3′; Mouse 3-MST: Forward 5′-CGAGACGGCATTGAACC-3,′ Reverse 5′-CTGGAACAGATGGCGGA-3′; Mouse Aldob: Forward 5′-GAAACCGCCTGCAAAGGATAA-3,′ Reverse 5′-GAGGGTCTCGTGGAAAAGGAT-3′; Mouse GLUT1: Forward 5′-TCAACACGGCCTTCACTG-3,′ Reverse 5′-CACGATGCTCAGATAGGACATC-3′; Mouse GLUT2: Forward 5′-ATTCGCCTGGATGAGTTACG-3,′ Reverse 5′-CAGCAACCATGAACCAAGG-3′; Mouse HK4: Forward 5′-TGAGCCGGATGCAGAAGGA-3,′ Reverse 5′-GCAACATCTTTACACTGGCCT-3′; Mouse PKM: Forward 5′-GCCGCCTGGACATTGACTC-3,′ Reverse 5′-CCATGAGAGAAATTCAGCCGAG-3′; Mouse PFKM: Forward 5′-TGTGGTCCGAGTTGGTATCTT-3,′ Reverse 5′-GCACTTCCAATCACTGTGCC-3′; Mouse Pfkfb2: Forward 5′-ACACTGTAATTTCTTGGACGCC-3,′ Reverse 5′-TGTGGTCCGAGTTGGTATCTT-3.′

S2.11 Flow cytometric analyses

We utilized the Annexin V/PI detection kit (C1062L, Beyotime, Shanghai, China) following the provided instructions to assess apoptosis levels in MIN6, β-TC-6, and MenSCs cell lines. Samples were analyzed using a CytoFLEX LX (Beckman Coulter, California, USA). For the identification of MenSCs surface markers we employed antibodies targeting CD117 (#561682), CD73 (#561014), CD90 (#561970), CD29 (#561795)，CD34 (#560941), CD45 (#560975), CD105 (#560839), and HLA-DR (#560943) procured from BD Biosciences (Franklin Lakes, NJ, USA). These samples were analyzed on a Novocyte flow cytometer (ACEA Biosciences, CA, USA).

S2.12 Immunofluorescence

The procedure was as described previously[32]. Primary antibodies against insulin (dilution: 1:400,#8138) were purchased from Cell Signaling Technology (Danvers, MA, USA). Goat anti-mouse IgG (H+L)-cross-adsorbed secondary antibodies and Alexa Fluor 488 (A11029) were purchased from Thermo Fisher Scientific (New York, USA). Hoechst33342 (C1025) was purchased from Beyotime Biotechnology (Shanghai, China). Images were captured using a Leica sp8 confocal laser scanning microscope (Leica, Germany).

S2.13 ELISA

All samples were collected and stored at -20°C in strict accordance with the manufacturer's instructions. Insulin levels

were measured using a Mouse Insulin ELISA kit (10-1247-01, Mercodia, Uppsala, Sweden) and a Mouse Insulin ELISA kit (RK09268, ABclonal, Wuhan, China). The level of interleukin6 (IL6) was measured using a Mouse IL-6 Fast ELISA Kit (RK04845; ABclonal, Wuhan, China). The levels of HK, PK, and PFK were measured using the Mouse Hexokinase ELISA Kit (MBS723284), Mouse PK ELISA Kit (MBS2509099), and Mouse Phosphofructokinase ELISA Kit(MBS9304835), respectively, which were purchased from Mybiosource (Hong Kong, China). All procedures were performed according to the manufacturer's instructions, and the absorbance of all samples on the ELISA plate was measured at 450 nm using a microplate reader (Molecular Devices, San Jose, California, USA).

S2.14 Immunohistochemistry

Immunohistochemistry was performed according to standardized procedures. Briefly, kidney specimens were initially fixed with paraformaldehyde, then underwent deparaffinization with xylene and dehydration using graded alcohol. This was followed by endogenous peroxidase blockade with hydrogen peroxide, antigen retrieval with citrate buffer, and subsequent blocking with goat serum. The specimens were subsequently incubated overnight with the following primary antibodies: anti-insulin (dilution: 1:6400,#3014, Cell Signaling Technology, Danvers, MA, USA), anti-CD31 (dilution: 1:200,#77699, Cell Signaling Technology, Danvers, MA, USA), and anti-Caspase3 (dilution: 1:100,ab184787, Abcam). The sections were then exposed to horseradish peroxidase (HRP)-conjugated secondary antibodies (Thermo Fisher Scientific) at room temperature (20-25° C) for 1 h. Images were captured using an OLYMPUS IX83-DP70 fluorescence microscope (Olympus Corporation).

S2.15 Statistical analysis

Differences among multiple groups were analyzed by one-way analysis of variance (ANOVA) with Tukey tests.. For statistical comparisons of the two groups, a Student's t-test was performed. The data are presented as means ± standard deviation (SD). Differences of at least $P < 0.05$ were considered significant, whereas p values ≥ 0.05 were considered non-significant. Statistical analyses were performed using GraphPad Prism 9 software (GraphPad, San Diego, CA, USA).
